# Eggshell translucency in late-phase laying hens and its effect on egg quality and physiological indicators

**DOI:** 10.3389/fvets.2023.1133752

**Published:** 2023-05-18

**Authors:** He-Ling Ren, Xiao-Yu Zhao, Ke-Qian Di, Lan-Hui Li, Er-Ying Hao, Hui Chen, Rong-Yan Zhou, Chang-Sheng Nie, De-He Wang

**Affiliations:** ^1^College of Animal Science and Technology, Hebei Agricultural University, Baoding, China; ^2^Baoding Xingrui Agriculture and Animal Husbandry Development Co., Ltd., Baoding, China; ^3^School of Basic Medical Sciences, Hebei University, Baoding, China; ^4^National Engineering Laboratory for Animal Breeding and MOA Key Laboratory of Animal Genetics and Breeding, College of Animal Science and Technology, China Agricultural University, Beijing, China

**Keywords:** late-phase laying hen, eggshell translucency, egg quality, physiological indicator, calcium

## Abstract

Eggshell translucency severely affects external egg quality, and variations in the eggshell or eggshell membrane are considered the structural basis of the trait. Research has shown that 1.85% additional mixed fatty acids in the diet would greatly decrease the occurrence of eggshell translucency. Only a few studies have examined the phenotypic regularity of eggshell translucency with the increasing age of hens. Therefore, two strains, 1139 Rhode Island Red-White (**RIR-White**) and 836 Dwarf Layer-White (**DWL-White**), were used, and from each strain, 30 hens each that consecutively laid translucent or opaque eggs at 67 wks of age were selected. Subsequently, eggshell translucency, internal quality and external quality of eggs, and total cholesterol, albumin, calcium binding protein and other physiological indicators related to lipid, lipoprotein, and calcium metabolisms at the 75^th^, 79^th^, and 83^rd^ wks of age in the late phase of the laying cycle were determined. Results: (1) In terms of flocks, for both strains, the translucency scores of the translucent groups were significantly higher than those of the opaque groups (*P* < 0.05); in terms of individuals, 81.1% RIR-White and 82.8% DWL-White hens consecutively laid eggs of the same or similar translucency, indicating the stability of the trait with increasing hen age; (2) In RIR-White, the eggshell strength of the translucent group at 75 weeks was significantly higher than that of the opaque group (*P* < 0.05); in DWL-White, the eggshell membrane thickness of the translucent group at the 75^th^ and 83^rd^ weeks was significantly lower than that of the opaque group (*P* < 0.05); (3) Compared to the opaque groups, the translucent groups had lower total cholesterol content in both RIR-White and DWL-White, lower albumin content in DWL-White at the 79^th^ weeks (*P* < 0.05), and higher calcium-binding protein (CALB1) in RIR-White at the 83^rd^ weeks (*P* < 0.05). In summary, this study illustrates the stability of eggshell translucency in late-phase laying hens and provides a reference of physiological indicators for exploring the formation of translucent eggs.

## Introduction

Eggshell translucency in hens was first reported in 1932 and is directly caused by penetration and accumulation of moisture in the eggshell (1, 2). Studies have shown that eggshell translucency is widely present in pure lines, commercial hens, and Chinese local strains, such as Rhode Island Red, White Leghorn, Brown-Egg Dwarf Layers, Hy-Line Brown hens, No.1 of Jing Hong laying hens, and Taihang chickens (2–4). In addition, compared with opaque eggs, the eggshell of translucent eggs is more easily penetrated by the Bifidobacterium and Escherichia coli, leaving a hidden danger to food safety (5). Traits of shell translucency are partly regulated by genetic factors, manifesting in differences in the severity of shell translucency among strains and between individuals. Furthermore, hens have laid eggs with the same level of translucency on consecutive days (3, 6). In previous studies, the heritabilities of eggshell translucency were estimated to be 0.18–0.22 in 63-wk-old Brown-Egg Dwarf Layers and a 52-wk-old F_2_ resource population generated from a cross between Dongxiang Blue-shelled and White Leghorn hens (2). Nevertheless, eggshell translucency is also partly affected by other factors, such as the temperature and humidity of the environment and the level of phosphorus in the diet (7). Additionally, eggshell translucency in older hens (60 weeks) appears to be more severe than that in younger hens (24 and 42 weeks) (8). However, the stability of the eggshell translucency trait in flocks and the continuity of the traits in individuals during the late phase of laying cycles has not been systematically investigated.

The structural basis of the translucent eggshell phenotype are variations of the eggshell's mineralized layer or eggshell membrane (2, 5, 8), of which the primary reason for eggshell translucency is yet to be confirmed. It has been reported that the eggshell thickness of translucent eggs was significantly higher than that of opaque eggs (9); however, certain studies have reported no difference between translucent and opaque eggs (7, 10). Furthermore, prominent, continuous grooves in the mammillary layer of eggshells and variations in mammillary caps were observed (5, 8); however, the results were not statistically analyzed. The eggshell is composed of 96% calcium, 2% organic matter, phosphorus, magnesium and other trace elements (11). Compared to hens laying opaque eggs, lower expression of Ca^2+^-ATPase mRNA was detected in the uterus tissues of hens laying translucent eggs, implying a low concentration of calcium ions in uterine fluids during eggshell formation (12). Scanning of normal eggshell ultrastructure using X-ray micro-computed tomography demonstrated that pore space accounts for almost one-third of the total eggshell volume (13). Bubble pores with 200–400 nm diameter are the main pore system in eggshells and play a paramount role in regulating gas exchange (14). However, bubble pores may also provide adequate space for moisture accumulation, resulting in eggshell translucency. In different studies, differences in eggshell thickness and strength were not consistent between translucent and opaque eggs (2, 7, 10). Furthermore, there was no difference in the quantity and size of bubble pores between translucent and opaque eggs (15), indicating that variations in the eggshell structure were important but may not be an essential factor affecting eggshell translucency.

The eggshell membrane is a fibrous network structure between the egg white and the mineralized eggshell layer. It not only provides a substrate for the initial mineralization during eggshell formation but also surrounds the egg contents and acts as a barrier, preventing egg contents from penetrating and accumulating in eggshells (11, 16). Therefore, the eggshell membrane may be an essential factor affecting eggshell translucency (2). Liu et al. (17) first discovered that the eggshell membrane of translucent eggs was thinner than that of opaque eggs. Further studies on the physical structure and chemical composition of the eggshell membrane demonstrated that the maximum longitudinal tensile break force and the total amino acid, proline, and lysine contents of the membrane in translucent eggs was significantly lower than those of opaque eggs (2), indicating that the contents of translucent eggs may penetrate through the eggshell membrane and accumulate in the eggshell more easily. Regarding nutrition, adding 1.85% mixed fatty acids, such as stearic acid, oleic acid, and linoleic acid, in the hens' diets for 4 weeks significantly reduced the percentage of translucent eggs from 15.9 to 4.4%, with no effect on the eggshell thickness and strength (18). Fatty acids may improve the quality of eggshell membrane by regulating the activity or expression of proteins and other substances involved in eggshell membrane formation through the lipid metabolism (19–21), thereby decreasing the percentage of translucent eggs.

The current study aimed to systematically investigate the characteristics of eggshell translucency in the late-phase laying cycle of hens, the stability of the translucency trait in the hen flock, and the continuity of shell translucency with the increasing age of the hens. We also compared the differences in the egg quality, blood lipid metabolism indicators, calcium metabolism related to the eggshell membrane, and the eggshell's mineralized layer between translucent and opaque eggs. These factors were chosen to provide a reference for determining the causes of eggshell translucency to help design strategies for reducing its occurrence.

## Materials and methods

### Experimental population and eggshell translucency identification

**Figure 1:**
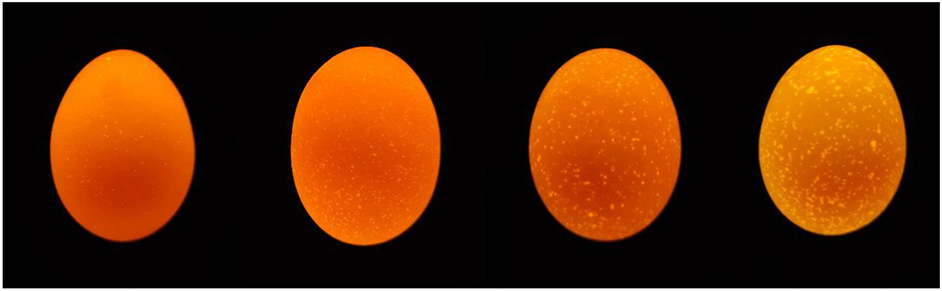
Reference samples used for the 1–4 scoring system (from left to right).

Sixty-seven-week-old hens of 1,139 Rhode Island Red-White (**RIR-White**) and 836 Dwarf Layer-White (**DWL-White**) strains were used in the experiment; all hens were raised in Hebei Rongde Poultry Breeding Co., Ltd. (Hengshui, China). Both RIR-White and DWL-White were bred for seven generations. RIR-White hens are white, lay brown-shelled eggs, have evolved from Rhode Island Red, and their body weight while laying the first egg is approximately 1.82 kg; DWL-White hens are white, lay pink-shelled eggs, and have evolved from Brown-Egg Dwarf Layers, which were developed by backcrosses of male meat-type dwarf ISA-Vedette to the female CAU brown egg layers (22). The two strains were reared in a fully enclosed underground house in individual cages (length 30 cm, width 40 cm, height 40 cm in front, and 33 cm in behind), with free access to food and water, a 16:8 h light-dark cycle, and automatic control of mechanical ventilation. Composition and nutrient levels of the basal diet are shown in Table 1. From each individual, we collected all eggs over five consecutive days and recorded the corresponding individual number. Meanwhile, abnormal eggs, such as broken, soft-shelled, or deformed eggs, were excluded. For each hen, if three qualified eggs were selected, we stopped collecting eggs from that hen in the next few days, and if less than three eggs were selected on the 5^th^ day, we also stopped collecting eggs. The collected eggs were stored in a stable environment (temperature: 20–25°C humidity: 40–50%) for 5 days and then classified based on their translucency levels (quantity and size of translucent spots on the eggshell) using a 1–4 scoring system (4). The reference sample used in the scoring method is shown in Figure 1. The higher the score, the more severe the eggshell translucency. The eggshell translucency level of each hen was determined by the average score value of 3 eggs. Finally, for each strain, 30 individuals with a higher eggshell translucency score and 30 individuals with a lower eggshell translucency score were selected and classified as translucent and opaque groups, respectively. Subsequently, the eggshell translucency levels of each hen were recorded at 75, 79, and 83 weeks of age; the method and process of eggshell translucency identification were the same as above.

**Table 1:** Composition and nutrient levels of the basal diet.

**Items**	**Content**
Ingredients (%)	
Corn	62.50
Soybean meal	25.20
Corn gluten meal	0.50
Rice bran	
Wheat bran	
Limestone	9.50
CaHPO4	0.90
DL-Met	0.10
Premix^a^	1.00
NaCl	0.30
Total	100.00
Nutrient levels^b^	
ME/(MJ/kg)	10.91
CP (%)	15.98
CF (%)	2.10
Ca (%)	3.63
P (%)	0.27
Lys (%)	0.80
Met (%)	0.45

^a^The premix provided the following per kg of the diet: VA 11 000 IU, VD_3_ 3 000 IU, VE 12 IU; VK 3 mg; VB_1_ 2 mg; VB_2_ 6 mg; VB_6_ 3 mg; VB_12_ 0.012 mg; biotin 0.04 mg; folic acid 0.6 mg; niacin 20 mg; pantothenic acid 10 mg; ethoxyquin 0.4 mg; Cu 10 mg; I 0.35 mg; Fe 65 mg; Mn120 mg; Se 0.25 mg; Zn 65 mg. ^b^Nutrient levels were all calculated values.

### Egg quality measurement

After eggs were stored for 7 d during the 75^th^, 79^th^, and 83^rd^ weeks, we randomly selected 30 eggs each from the translucent and opaque groups of both RIR-White and DWL-White strains and measured their egg quality. The indicators studied were as follows: egg weight, 7-d water loss, egg shape index, eggshell thickness, eggshell membrane thickness, eggshell strength, Haugh unit, yolk color, eggshell weight, eggshell proportion, egg white weight, and yolk weight. Egg weight, yolk color, and Haugh unit were measured using an egg quality tester (EA-01; Israel Aoke Co., Ltd., Israel). The 7-d water loss, eggshell weight, egg white weight, and yolk weight were measured using an electronic analytical balance (PL1502-S; Mettler Toledo Instruments Co., LTD., Switzerland). The 7-d water loss rate is the ratio of 7-d water loss to initial egg weight; eggshell proportion is the ratio of eggshell weight to egg weight; the eggshell was first washed using deionized water to remove albumen and left for 30 min to dry naturally. Then the eggshells were weighed using an electronic analytical balance as above. Egg shape index is the ratio of the long diameter of an egg to its short diameter and was measured using an NFN384 egg quality analyzer (FHK, Tokyo, Japan). Eggshell thickness and eggshell membrane thickness were measured using digital vernier calipers (MNT-LG11-121-1; Guangzhou Lige Technology Co., Ltd., Guangzhou); eggshell membrane thickness is the value of the total eggshell thickness minus the thickness of the mineralized eggshell layer. Eggshell strength was determined using an eggshell strength tester (ESTG-01; Israel Aoke Co., Ltd., Israel).

### Plasma biochemistry

On the 67^th^ of the 75^th^, 79^th^, and 83^rd^ wks, after completing the egg collection procedure, we collected 3 mL of inferior pterygoid venous blood from each hen and put it into an anticoagulant tube with heparin sodium as an anticoagulant (Shandong Junnuo Medical Devices Co., Ltd., Shandong, China). Plasma samples were prepared by centrifugation at 3,000 rpm for 15 min (LG10-2.4A; Beijing Rebel centrifuge Co., Ltd., China). The glycerine phosphate oxidase peroxidase (GPO-PAP) method (23) and the cholesterol oxidase phenol 4-aminoantipyrine peroxidase (CHOD-PAP) assay (24) were used to measure triglyceride and total cholesterol contents, respectively. The contents of total protein were detected by bicinchoninic acid (BCA) assay (25) and albumin was detected by bromocresol green (BCG) assay (26), while the methylthymol blue (MTB) method (27), and the molybdenum blue method (28) were used to determine the calcium and phosphorus levels, respectively (Nanjing Jiancheng Bioengineering Institute, Nanjing, China). The globulin content is the difference between the total protein and albumin contents. The contents of estrogen, calcium-binding protein (CALB1), and calcium pump were measured using double-antibody sandwich enzyme-linked immunosorbent assay (ELISA) (29) kits (Shanghai Jianglai Biotechnology Co., Ltd., Shanghai, China).

### Statistical analysis

Using non-parametric Mann-Whitney tests (30), the ordinal shell translucency score data per hen strain were compared at different weeks of age between translucent and opaque groups, as well as across age within each group. All egg quality and blood indicators of the translucent and opaque groups at the same week were compared using a *t*-test (all data were normally distributed). The between groups variance of egg quality and blood indicators among the different weeks of hen age was analyzed using SPSS software (version 23.0, SPSS Inc., Chicago, IL) in the general linear model (GLM), and the model used is shown in the following equation:


$$\begin{array}{l} X_{ij}=\mu +\alpha_{i}+e_{ij} \end{array}$$

where *X*_*ij*_ is the measurement value of the different indicators, μ is the overall average, α_*i*_ is the effect of groups, and *e*_*ij*_ is the residual error. Differences in the average values among groups for each indicator were compared using Duncan's multiple range test. The significance level was set at 0.05.

## Results

### Eggshell translucency

Variations in eggshell translucency with the increase in hen age of both RIR-White and DWL-White strains are shown in Table 2. For RIR-White, in the 67^th^ wk, the mean eggshell translucency score of the translucent group (3.34) was significantly higher than that of the opaque group (1.93; *P* < 0.05). In the 75^th^, 79^th^, and 83^rd^ weeks, the trend was consistent with that of the 67^th^ wk; the mean translucency scores of the translucent group were still significantly higher than those of the opaque group (*P* < 0.05). For DWL-White, the mean translucency score of the translucent group at the 67^th^ wk was 3.19, which was significantly higher than that of the opaque group (1.92; *P* < 0.05). In the 75^th^, 79^th^, and 83^rd^ weeks, the results were consistent with those of RIR-White; mean translucency scores of the translucent group were still significantly higher than those of the opaque group (*P* < 0.05). For the translucent group, the translucency score of the 83^rd^ wk was significantly lower than those of the 67^th^ weeks in RIR-White (*P* < 0.05). For the opaque group, the translucency score of the 67^th^ wk was significantly lower than those of 75^th^ and 79^th^ weeks in the DWL-White strain (*P* < 0.05). In RIR-White strain, the average translucency score of the translucent group decreased with hen age, and in DWL-White strain, the average translucency score of the opaque group increased with hen age.

**Table 2:** The eggshell translucency of different hen strains in the late-phase laying cycle.

**Strain**	**Week**	**Number of eggs/hen**		**Score of shell translucency**	
		**Translucent**	**Opaque**	**Translucent**	**Opaque**
Rhode Island Red-White (*N =* 116)	67	2.46 ± 0.90	2.61 ± 0.67	3.34 ± 0.46^ax^	1.93 ± 0.40^z^
	75	2.60 ± 0.93	2.81 ± 0.60	3.21 ± 0.71^abx^	2.21 ± 0.57^z^
	79	2.16 ± 1.14	2.33 ± 0.91	3.12 ± 0.66^abx^	2.58 ± 0.96^z^
	83	2.22 ± 1.31	2.19 ± 1.29	2.86 ± 0.72^bx^	2.08 ± 0.75^z^
Dwarf Layer-White (*N =* 116)	67	2.41 ± 0.84	2.69 ± 0.55	3.19 ± 0.51^x^	1.92 ± 0.32^az^
	75	2.78 ± 0.61	2.73 ± 0.72	3.04 ± 0.75^x^	2.57 ± 0.71^bz^
	79	2.16 ± 1.17	2.46 ± 0.99	3.07 ± 0.74^x^	2.43 ± 0.64^bz^
	83	2.50 ± 1.11	2.35 ± 1.09	2.77 ± 0.72^x^	2.26 ± 0.74^abz^

^a, b^Within each column, there were statistical differences in the score of shell translucency among the same strain at different weeks of age. ^x, z^Within each row, there are statistical differences in the score of shell translucency among the same strain for translucent and opaque groups per age week. The score of shell translucency was per a 1–4 scoring system (score 4 is most translucent). All values are mean ± standard deviation.

**Figure 2:**
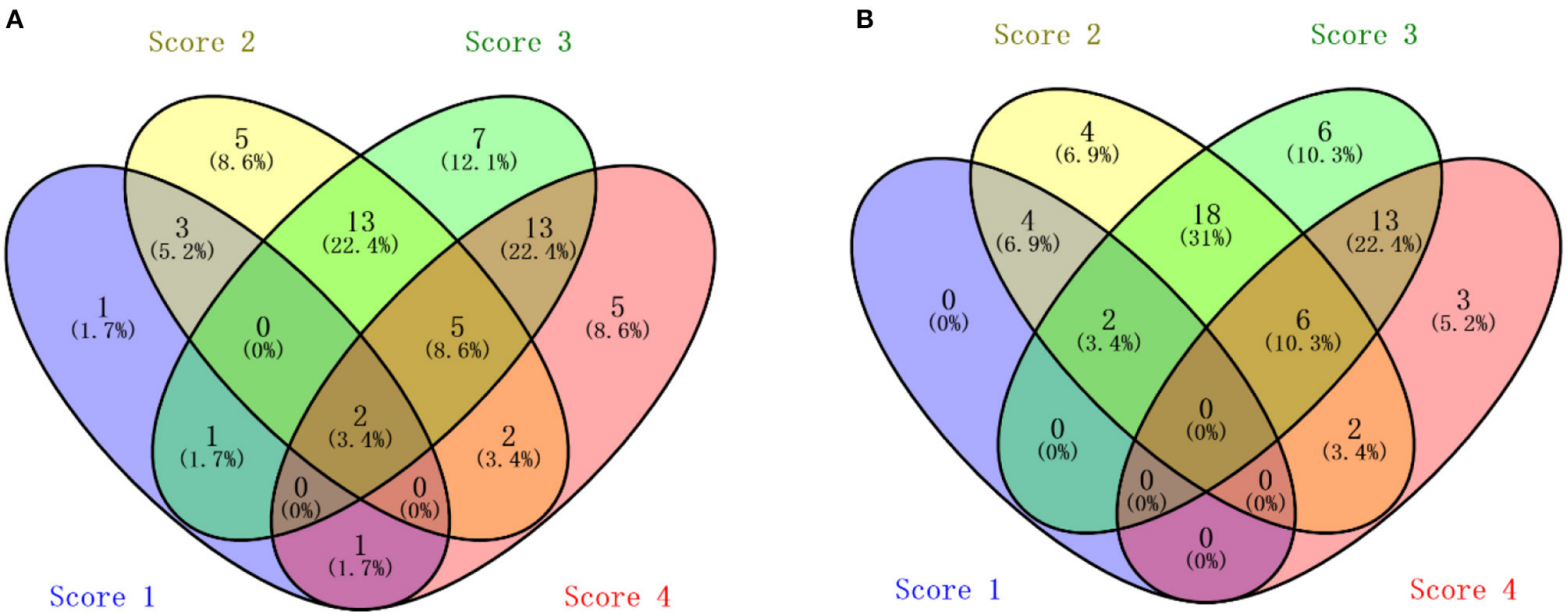
The Venn diagram of average scores of eggshell translucency at 67^th^, 75^th^, 79^th^, and 83^rd^ weeks in 2 strains. **(A)** Venn diagram of Rhode Island Red-White; **(B)** Venn diagram of Dwarf Layer-White.

Regarding individuals, variations of eggshell translucency with hen age are presented in Figure 2. In RIR-White, 81.1% of hens successively laid eggs of the same or adjacent translucency scores on the 67^th^, 75^th^, 79^th^, and 83^rd^ weeks. Among these, 31% (18 individuals) of hens successively laid eggs of the same score (1.7, 8.6, and 12.1%, 8.6% of hens laid eggs of scores 1, 2, 3, and 4, respectively); 50% (29 individuals) of hens laid eggs of adjacent scores (5.2% of hens laid eggs of score 1 and 2, 22.4% of hens laid eggs of score 2 and 3, and 22.4% of hens laid eggs of scores 3 and 4); 18.9% (11 individuals) of hens laid eggs covering over three score levels. In DWL-White, similar to RIR-White, 82.8% of hens laid eggs of the same or adjacent translucency scores. Among these, 22.4% (13 individuals) of hens laid eggs of the same score, 60.3% (35 individuals) of hens laid eggs of adjacent translucency scores, (6.9% of hens laid eggs of scores 1 and 2, 31% of hens laid eggs of scores 2 and 3, and 22.4% of hens laid eggs of scores 3 and 4), and 17.2% (10 individuals) of hens laid eggs covering over three score levels.

### Egg quality

Detailed metrics of egg quality of the translucent and opaque groups of both RIR-White and DWL-White strains are shown in Tables 3, 4. In RIR-White, the egg weight, eggshell strength, and eggshell weight of the translucent group were significantly higher than those of the opaque group at 75 weeks of age (*P* < 0.05), whereas the eggshell thickness tended to be higher than that of the opaque group. In 83-wk-old hens, the egg weight, egg white weight, and yolk weight of the translucent group were significantly higher, and the 7-d water loss rate was significantly lower than those of the opaque group (*P* < 0.05), whereas the eggshell strength of the translucent group tended to be higher than that of the opaque group. In DWL-White, the eggshell membrane thickness of the translucent group was significantly thinner than that of the opaque group at 75 and 83 weeks of age (*P* < 0.05), and there were no significant differences in other indicators. In the 79^th^ wk of age, the yolk color of the translucent group was significantly higher than that of the opaque group in RIR-White (*P* < 0.05), whereas the opposite was true in DWL-White.

**Table 3:** Comparison of egg quality between translucent and opaque groups of Rhode Island Red-White across age.

**Traits/age**	**75 week**		**79 week**		**83 week**	
	**Translucent**	**Opaque**	**Translucent**	**Opaque**	**Translucent**	**Opaque**
Translucent grade	3.21 ± 0.71^a^	2.21 ± 0.57^b^	3.12 ± 0.66^a^	2.58 ± 0.96^b^	2.86 ± 0.72^a^	2.08 ± 0.75^b^
Egg weight (g)	62.87 ± 3.85^a^	60.18 ± 4.02^b^	61.29 ± 5.15	59.58 ± 5.26	63.21 ± 3.88^a^	60.14 ± 5.13^b^
Water loss rate (%)	1.83 ± 0.48	1.69 ± 0.40	1.17 ± 0.33	1.06 ± 0.30	2.08 ± 0.41^a^	2.41 ± 0.37^b^
Egg shape index	1.37 ± 0.05	1.36 ± 0.04	1.37 ± 0.04	1.35 ± 0.04	1.35 ± 0.07	1.38 ± 0.06
Eggshell strength (N)	33.68 ± 6.72^a^	28.14 ± 6.78^b^	30.29 ± 6.16	30.43 ± 6.32	28.81 ± 7.06	26.71 ± 6.47
Eggshell thickness (μm)	325.20 ± 22.56	318.00 ± 28.42	323.14 ± 25.28	321.00 ± 28.21	319.42 ± 29.09	318.13 ± 29.14
Eggshell membrane thickness (μm)	13.48 ± 3.96	12.19 ± 4.26	17.93 ± 6.79	17.33 ± 5.17	11.69 ± 4.11	10.40 ± 3.69
Eggshell weight (g)	7.81 ± 0.63^a^	7.26 ± 0.51^b^	7.84 ± 0.61	7.71 ± 0.89	7.58 ± 0.56	7.37 ± 0.53
Eggshell proportion (%)	12.43 ± 1.12	12.02 ± 1.00	12.63 ± 0.74	12.96 ± 0.99	11.98 ± 0.69	12.12 ± 0.72
Haugh unit	82.14 ± 3.99	82.40 ± 2.54	76.99 ± 3.07	77.71 ± 4.61	82.23 ± 4.37	80.12 ± 5.61
Egg white weight (g)	36.21 ± 3.14	35.34 ± 3.85	35.57 ± 3.51	34.74 ± 3.90	35.75 ± 2.79^a^	33.52 ± 4.16^b^
Yolk color	9.90 ± 1.25	9.57 ± 1.32	11.21 ± 0.57^a^	10.60 ± 0.94^b^	11.63 ± 0.49	11.60 ± 0.51
Yolk weight (g)	17.57 ± 1.34	17.04 ± 1.22	17.76 ± 1.48	17.58 ± 1.77	18.81 ± 1.39^a^	17.65 ± 1.66^b^

All values are mean ± standard deviation. ^a, b^Within each row, means values with different letters indicate statistical differences between translucent groups and opaque groups of the same week (*P* < 0.05).

**Table 4:** Comparison of egg quality between two groups of Dwarf Layer-White across age.

**Traits/age**	**75 week**		**79 week**		**83 week**	
	**Translucent**	**Opaque**	**Translucent**	**Opaque**	**Translucent**	**Opaque**
Translucent grade	3.17 ± 0.72^a^	2.43 ± 0.67^b^	3.07 ± 0.75^a^	2.43 ± 0.64^b^	2.77 ± 0.72^a^	2.26 ± 0.74^b^
Egg weight (g)	56.87 ± 3.63	55.70 ± 5.12	57.94 ± 4.97	56.82 ± 6.22	57.78 ± 5.81	57.98 ± 5.52
Water loss rate (%)	1.92 ± 0.51	1.74 ± 0.42	1.16 ± 0.33	1.23 ± 0.30	2.11 ± 0.67	1.88 ± 0.49
Egg shape index	1.36 ± 0.05	1.36 ± 0.51	1.39 ± 0.52	1.36 ± 0.45	1.35 ± 0.06	1.34 ± 0.07
Eggshell strength (N)	31.45 ± 7.94	32.78 ± 4.64	31.74 ± 6.97	31.56 ± 6.95	28.22 ± 6.89	26.65 ± 6.72
Eggshell thickness (μm)	316.06 ± 28.34	316.69 ± 32.51	310.91 ± 26.00	316.04 ± 26.82	307.60 ± 26.88	312.50 ± 27.49
Eggshell membrane thickness (μm)	11.68 ± 3.15^a^	14.32 ± 4.70^b^	13.83 ± 5.24	13.68 ± 3.78	9.78 ± 2.73^a^	12.52 ± 4.59^b^
Eggshell weight (g)	7.32 ± 0.46	7.05 ± 0.53	7.25 ± 0.55	7.35 ± 0.68	7.02 ± 0.67	7.06 ± 0.56
Eggshell proportion (%)	12.79 ± 0.60	12.73 ± 1.12	12.25 ± 0.99	12.49 ± 0.82	11.93 ± 1.00	12.09 ± 0.99
Haugh unit	79.55 ± 5.16	80.17 ± 3.32	77.42 ± 5.55	76.38 ± 3.66	80.14 ± 5.61	81.07 ± 4.68
Egg white weight (g)	32.17 ± 3.73	30.81 ± 3.62	32.27 ± 4.20	31.61 ± 4.19	31.21 ± 4.73	31.40 ± 3.47
Yolk color	9.17 ± 1.14	9.52 ± 1.31	10.60 ± 0.87^a^	11.22 ± 0.90^b^	10.00 ± 0.96	9.86 ± 0.99
Yolk weight (g)	17.02 ± 1.35	16.87 ± 1.46	17.82 ± 1.42	17.13 ± 1.81	18.51 ± 1.91	18.00 ± 1.55

All values are mean ± standard deviation. ^a, b^Within each row, means values with different letters indicate statistical differences between translucent groups and opaque groups of the same week (*P* < 0.05).

### Blood indicators

The results for all blood indicator analyses are presented in Tables 5, 6. In RIR-White, at the 75^th^ wk, the total cholesterol of the translucent group was significantly lower than that of the opaque group (*P* < 0.05). In DWL-White, similar results were obtained at the 79^th^ and 83^rd^ weeks; the total cholesterol of the translucent group was significantly lower than those of the opaque group (*P* < 0.05), and the same trend was also observed at the 75^th^ wk. At the 83^rd^ wk in RIR-White, the CALB1 levels of the translucent group were significantly higher than those of the opaque group (*P* < 0.05) and the 79^th^ wk in DWL-White found the same trend. Furthermore, in RIR-White, at the 83^rd^ wk, the phosphorus content of the translucent group was significantly lower than that of the opaque group (*P* < 0.05). In DWL-White, the albumin content of the translucent group was significantly lower than that of the opaque group at the 79^th^ week of age, whereas the estrogen content of the translucent group was significantly higher than that of the opaque group at the 75^th^ week. There were no differences in other indicators.

**Table 5:** Comparison of plasma biochemistry indexes between translucent and opaque groups of Rhode Island Red-White.

**Traits/age**	**75 week**		**79 week**		**83 week**	
	**Translucent**	**Opaque**	**Translucent**	**Opaque**	**Translucent**	**Opaque**
Triglyceride (mmol/L)	16.44 ± 7.74	15.50 ± 7.14	17.54 ± 8.44	14.05 ± 6.05	27.71 ± 6.79	27.63 ± 7.76
Total cholesterol (mmol/L)	4.11 ± 1.42^a^	4.93 ± 1.15^b^	3.78 ± 0.73	4.07 ± 1.48	3.78 ± 0.83	4.39 ± 1.16
Total protein (g/L)	131.94 ± 27.67	137.6 ± 33.72	178.29 ± 29.02	173.81 ± 31.19	226.93 ± 43.33	216.72 ± 42.24
Albumen (g/L)	33.86 ± 10.48	37.98 ± 12.69	28.92 ± 4.24	29.06 ± 3.51	24.30 ± 4.62	22.60 ± 2.86
Globulin (g/L)	93.38 ± 29.63	100.62 ± 27.87	148.67 ± 28.91	144.02 ± 31.32	202.76 ± 42.60	193.32 ± 41.72
Ca (mmol/L)	1.88 ± 0.29	1.87 ± 0.21	1.45 ± 0.09	1.49 ± 0.12	1.48 ± 0.44	1.48 ± 0.50
P (mmol/L)	1.39 ± 0.33	1.37 ± 0.39	1.54 ± 0.33	1.56 ± 0.29	1.68 ± 0.26^a^	1.97 ± 0.44^b^
Calcium pump (μmol/mL)	11.29 ± 1.75	11.29 ± 2.11	11.89 ± 2.17	12.12 ± 2.21	10.83 ± 2.48	11.51 ± 2.59
Calcium binding protein (ng/mL)	6.13 ± 0.98	5.98 ± 0.87	6.43 ± 1.50	6.34 ± 1.26	7.23 ± 1.47^a^	5.99 ± 1.39^b^
Estrogen (pg/mL)	58.32 ± 10.68	55.63 ± 12.30	62.36 ± 12.62	66.70 ± 13.53	49.01 ± 9.78	54.34 ± 11.98

All values are mean ± standard deviation. ^a, b^Within each row, means values with different letters indicate statistical differences between translucent groups and opaque groups of the same week (*P* < 0.05).

**Table 6:** Comparison of plasma biochemistry indexes between translucent and opaque groups of Dwarf Layer-White.

**Traits/age**	**75 week**		**79 week**		**83 week**	
	**Translucent**	**Opaque**	**Translucent**	**Opaque**	**Translucent**	**Opaque**
Triglyceride (mmol/L)	14.47 ± 7.77	13.83 ± 6.67	17.02 ± 4.17	16.40 ± 5.45	32.03 ± 8.85	32.27 ± 5.91
Total cholesterol (mmol/L)	4.99 ± 1.80	5.38 ± 1.69	5.82 ± 2.58^a^	7.65 ± 3.23^b^	4.37 ± 1.34^a^	5.72 ± 2.28^b^
Total protein (g/L)	169.11 ± 38.17	183.86 ± 49.18	161.62 ± 38.00	159.51 ± 40.73	257.54 ± 42.98	237.71 ± 45.19
Albumen (g/L)	45.23 ± 14.62	60.41 ± 30.88	29.56 ± 3.16^a^	34.36 ± 5.32^b^	24.24 ± 4.05	25.18 ± 3.33
Globulin (g/L)	114.75 ± 34.72	122.45 ± 37.58	127.52 ± 35.81	116.74 ± 34.64	227.65 ± 45.39	216.86 ± 41.65
Ca (mmol/L)	1.96 ± 0.31	2.09 ± 0.37	1.62 ± 0.15	1.66 ± 0.266	1.54 ± 0.14	1.62 ± 0.16
P (mmol/L)	1.61 ± 0.37	1.46 ± 0.43	1.47 ± 0.37	1.77 ± 0.73	1.77 ± 0.38	1.83 ± 0.56
Calcium pump (μmol/mL)	11.71 ± 1.56	11.13 ± 2.53	12.01 ± 1.67	12.16 ± 2.20	12.16 ± 2.38	11.76 ± 2.67
Calcium binding protein (ng/mL)	6.20 ± 1.09	6.36 ± 1.28	7.09 ± 1.59^a^	6.33 ± 0.75^b^	6.75 ± 1.44	7.06 ± 1.08
Estrogen (pg/mL)	62.94 ± 10.40^a^	55.53 ± 9.34^b^	65.71 ± 9.93	66.08 ± 10.80	47.05 ± 12.09	48.77 ± 10.09

All values are mean ± standard deviation. ^a, b^Within each row, means values with different letters indicate statistical differences between translucent groups and opaque groups of the same week (*P* < 0.05).

## Discussion

### Eggshell translucency

This experiment tracked the eggshell translucency of RIR-White and DWL-White strains at the 67^th^, 75^th^, 79^th^, and 83^rd^ weeks of age. In terms of the hen flock, consistent differences were observed in the average eggshell translucency score between the translucent and opaque groups in both strains from the 67^th^ to 83^rd^ week, implying the trait stability of eggshell translucency. With the increase in the age of the hens, the difference in shell translucency between the translucent and opaque groups became smaller. Shell translucency of the opaque group tended to be more severe with the increasing age of hens, whereas shell translucency of the translucent group tended to be less with the increasing age of hens; these observations are consistent with those of Solomon (8). Tables 3, 4 show that for both strains, the eggshell strength and thickness of the translucent groups tended to be lower and thinner (31), respectively, with the increase in hens' age. It is speculated that variations in the eggshell may affect the conditions for water accumulation in the eggshell (13), resulting in the decrease of shell translucency. However, the translucent group maintained a high score for eggshell translucency, suggesting that variations in the eggshell may not be the essential cause for eggshell translucency.

Regarding individual hens, 81% RIR-White and 82.7% DWL-White hens laid eggs of the same or adjacent eggshell translucency score from the 67^th^ to 83^rd^ wk, indicating the stability of the eggshell translucency trait in late-phase laying hens. Measurement error may be an important reason for the occurrence of adjacent scores. When using the scoring method, an egg with a specific score of shell translucency has an approximately 50% probability of being misclassified into the adjacent score (4). Another reason may be that the shell translucency phenotype did change slightly (Table 2), and these changes may be caused by non-essential factors for the occurrence of shell translucency. Above all, the results correspond with previous studies suggesting that hens laid translucent eggs on consecutive days (2, 6) and prove the trait stability of eggshell translucency in the late-phase laying cycle of hens.

### Egg quality

In DWL-White hens, the eggshell membrane thickness of the translucent group was significantly lower than that of the opaque group, and this was consistent with the thinner eggshell membrane and lower maximum tensile break strength of the translucent group reported in Brown-Egg Dwarf Layers (2). Translucent spots on the eggshell were the result of accumulation and uneven distribution of moisture from egg contents (1). The eggshell membrane is a complex network of fibers that wrap egg contents and act as a barrier for preventing egg contents from penetrating the eggshell (11, 16). However, the eggshell membrane is also porous, and the porosity was estimated to be 52.06% using an atomic force microscope (32). During storage, changes in the environmental temperature and the release of carbon dioxide in egg whites (33) may cause changes in the volume of egg contents, promoting the rupture of thinner or less tough eggshell membranes, allowing egg contents and moisture to penetrate the eggshell membrane and accumulate in the eggshell. However, because there is no difference in pore distribution between translucent eggs and opaque eggs, more water can gather in the eggshell, forming translucent eggs (34).

In RIR-White, at the 75^th^ wk, the eggshell strength of the translucent group was significantly higher than that of the opaque group, and the eggshell thickness at the 75^th^ wk and eggshell strength at the 83^rd^ wk also showed the same trend. These results are consistent with previous research results in White Leghorn layer hens and Brown-Egg Dwarf Layers (9). Eggshell strength is directly related to the eggshell thickness (35), crystal ultrastructure of the eggshell (36), and the attachment strength between the eggshell membrane and mammillary layer (37). In 40-wk-old Isa Brown laying hens, it was reported that the cap structure of mammillary cores in the translucent group was more fully in contact with the eggshell membrane (5), contributing to the enhancement of eggshell strength and eggshell thickness (38). In the current study, the differences in eggshell and eggshell membrane between the translucent and opaque groups of the two strains were not entirely consistent. But the results do not conflict with the fact that the shell membrane of translucent eggs is thinner (7), the eggshell strength is higher (2), and the eggshell thickness is higher (39). One important reason for these results may be the thinner eggshell membrane in the late-phase laying cycle of the two strains, with average values of approximately 11–13 μm, which were lower than those in White Leghorn and Brown-Egg Dwarf Layers (18–21 μm) (9). We speculate that a thinner eggshell membrane may be a prerequisite for eggshell translucency.

Most studies have shown no difference in protein height, haugh unit, yolk weight and in other internal egg quality measurements between translucent and opaque groups (1, 40). However, in this study, in RIR-White, the egg weight, eggshell weight, egg yolk weight, and egg white weight of the translucent group at the 75^th^ or 83^rd^ week were higher than those of the opaque group, and the water loss rate was lower than that of the opaque group. Among these parameters, egg weight is mainly affected by body weight (41), indicating that the physiological status of layers were all affected by the eggshell translucency to a certain extent. Eggshell weight, yolk weight, and egg white weight was all affected by egg weight, which was positively correlated with egg weight (42). In addition, the smaller the egg weight, the larger the surface area/volume ratio (43), which may be responsible for the higher water loss rate in the opaque group.

### Plasma biochemistry

Studies have shown that including 1.85% of mixed fatty acids in hens' diets decreases eggshell translucency but does not affect eggshell strength and thickness (18); however, it may improve the quality of the eggshell membrane. Total cholesterol is an important indicator of blood lipid metabolism (44). In this experiment, the total cholesterol of the translucent group was lower than that of the opaque group, and this corresponded with the fact that the candidate gene for the translucency trait, Acetyl-CoA carboxylase alpha (ACACA), was associated with abdominal fat through epistatic effects (2, 45). During egg formation, when eggs travel through the isthmus of the fallopian tube, the expression of proteins such as collagen X, fibrillin-1 (FBN1), lysyl oxidase (LOX), and sulfhydryl oxidase 1 (QSOX1) play important roles in the formation of eggshell membranes (46). FBN1 affects the elasticity of eggshell membranes (46), and the expression of FBN1 is significantly up-regulated in individuals with high levels of body fat (20). In addition, LOX activity was overactivated in lipopolysaccharide-treated rat lungs (47), and the expression of the LOX family genes in the oviduct affected the structure of the eggshell membrane by promoting the connection between fibrous protein and collagen in the eggshell membrane (21). In DWL-White, the albumin content of the opaque group was higher than that of the translucent group. It has been reported that elevated plasma albumin would promote wound repair of liver cells (48) and that lipids, lipoproteins, and proteins synthesized by the liver are important precursors involved in egg formation (49). Thus, it is hypothesized that lipid synthesis disorder in the liver may affect eggshell membrane formation, resulting in a thinner eggshell membrane and causing eggshell translucency.

In both RIR-White and DWL-White, higher levels of CALB1 were identified in the translucent group, and a higher estrogen level was also observed in the translucent group of DWL-White at the 75^th^ week. Since both higher levels of CALB1 and estrogen promote calcium deposition during eggshell formation (12, 50, 51), the observations are consistent with the higher eggshell strength in the translucent group. The CALB1 in the intestine and uterus tissue of chickens is related to the absorption and secretion of calcium ions (50, 51). In the duodenum, the expression of the CALB1 decreased when hens laid soft-shelled eggs and increased when hens laid normal-shelled eggs (50, 51), whereas the concentration of CALB1 in the uterine tissue was regulated by estrogen levels (51). In addition, estrogen could also stimulate osteoblasts to produce the medullary bone (50), and the decomposition and reconstruction of medullary bone is closely related with eggshell formation (50, 51). However, in the current study, the expression of CALB1 in the translucent group contradicts with previous research (7, 10), which is probably because in the studies by Nie (7) and Jiang (10), the differences in eggshell and shell membrane were not identified. In RIR-White, the inorganic phosphorus content of the opaque group at the 83^rd^ wk was significantly higher than that of the translucent group, and this observation is consistent with the observation by Nie (7).

## Conclusion

In RIR-White and DWL-White, for hen individuals and flocks, the trait of eggshell translucency is stable during the late-phase laying cycle (from the 67^th^ to 83^rd^ week), indicating the feasibility to solve eggshell translucency from a genetic perspective. For eggshell and eggshell membrane, the eggshell strength and thickness of the translucent group tended to be higher than those of the opaque group, and the eggshell membrane thickness tended to be thinner than that of the opaque group. While both eggshell and eggshell membrane contributed to formation of eggshell translucency, the eggshell membrane may play a more important role. For physiological indicators, the total cholesterol and albumin contents of the translucent group tended to be lower than those of the opaque group. The CALB1 and estrogen levels of the translucent group tended to be higher than those of the opaque group. In the two strains of RIR-White and DWL-White, parts of egg quality and physiological indicators of translucent and opaque hen group are not completely consistent, which may caused by characteristics of hen strain or random errors. This study provides reference physiological indicators for exploring the formation of translucent eggs from a genetic perspective.

## Data availability statement

The raw data supporting the conclusions of this article will be made available by the authors, without undue reservation.

## Ethics statement

All procedures used in this study were approved by the Animal Use and Ethical Committee of Hebei Agricultural University (University Identification No. HB/2019/03). Written informed consent was obtained from the owners for the participation of their animals in this study.

## Author contributions

Main experimental methods: D-HW, L-HL, and R-YZ. Writing: H-LR. Operation: X-YZ. Supervision: K-QD, HC, and C-SN. Manuscript modification: D-HW and E-YH. All authors read and approved the final manuscript.
